# The great outdoors: how a green exercise environment can benefit all

**DOI:** 10.1186/2046-7648-2-3

**Published:** 2013-01-03

**Authors:** Valerie F Gladwell, Daniel K Brown, Carly Wood, Gavin R Sandercock, Jo L Barton

**Affiliations:** 1 School of Biological Sciences, University of Essex, Colchester, CO4 3SQ, UK

**Keywords:** Green exercise, Physiology, Natural environment, Physical activity, Motivation, Health

## Abstract

The studies of human and environment interactions usually consider the extremes of environment on individuals or how humans affect the environment. It is well known that physical activity improves both physiological and psychological well-being, but further evidence is required to ascertain how different environments influence and shape health. This review considers the declining levels of physical activity, particularly in the Western world, and how the environment may help motivate and facilitate physical activity. It also addresses the additional physiological and mental health benefits that appear to occur when exercise is performed in an outdoor environment. However, people’s connectedness to nature appears to be changing and this has important implications as to how humans are now interacting with nature. Barriers exist, and it is important that these are considered when discussing how to make exercise in the outdoors accessible and beneficial for all. The synergistic combination of exercise and exposure to nature and thus the ‘great outdoors’ could be used as a powerful tool to help fight the growing incidence of both physical inactivity and non-communicable disease.

## Review

### Background

Most discussions of human interactions with the environment concern the potential challenges they place on one another. These usually concern the extreme environmental demands such as those seen at high altitude, at depth or in extremes of temperature. Alternatively, they express the growing human population’s ongoing tendency to negatively influence the delicate balance of nature, which developed for millions of years prior to our evolutionary invasion.

With the multiplicity of the ‘great outdoors’ including forests, seaside, countryside, parks, local green areas and even gardens, another conversation considers the role of environment in benefiting human health. Green or natural spaces have been considered to be advantageous for health for many years. For example, in the UK during the 19th century Industrial Revolution, wealthy philanthropists developed urban parks for the benefit of the public’s health, and hospital gardens were considered an important addition for their believed healing properties [1,2]. A study in the early 21st century has further supported this belief, demonstrating an association between improved health outcomes and amount of surrounding ‘green space’ [3,4]. Subsequently, how and why the great outdoors may elicit health benefits has become a focal point for research.

Our hunter-gatherer ancestors existed with the outdoor natural environment for thousands of years, and it is hypothesised that this provides present day humans with an innate affiliation with nature [5]. In addition, nature provides an environment that does not require our direct attention, giving nature restorative properties therefore allowing recovery from mental fatigue [6] and attention restoration [7]. Although in the Western world, less people are involved in the natural environment on a daily basis, in particular reduced numbers working on the land, many people seek out nature and undertake outdoor recreational activities. Currently, there is an increasing trend for people to undertake outdoor endurance challenges but, paradoxically, there is a greater proportion of the population with insufficient physical activity levels to meet current health guidelines [8]. Recent reviews indicate that exercising outdoors appears to be more beneficial to mental health over indoor activities [9] and furthermore, natural environments have a greater impact on psychological health especially when exercise is incorporated [10]. To describe this potential synergistic benefit to health that occurs when exercising whilst being exposed to nature, the term ‘green exercise’ was adopted in 2003 [11] and published through peer-review in 2005 [12].

Pretty et al. [11] demonstrated that green exercise can improve mental well-being and markers of physiological health. There is subsequent preliminary evidence at physiological [13-15], psychological [9,16,17], biochemical [18] and social levels [17], which suggests that green exercise might have a useful role in primary and secondary prevention of disease. Moreover, there is evidence to suggest a role for green exercise in rehabilitation programmes [17]. In addition, engaging sedentary individuals in green exercise could be an effective vehicle in driving behavioural change by improving adherence rates to exercise programmes [19]. There is still a need to investigate the mechanisms behind observed health benefits of the natural environment [9,10]. A greater understanding of how nature positively interacts with human socio-biology may be mutually beneficial to both health and the environment.

The focus of this paper is to identify literature regarding physiological changes which occur as a product of participating in green exercise. Additionally, the likely interaction between these physiological changes and the well-documented psychological alterations will be discussed with regard to their potential health benefits. Furthermore, the impact that the great outdoors may have on exercise adherence and motivation to exercise will be explored in the context of increasing physical activity levels. Therefore the aims of the review are the following:

1. Outline the declining physical activity levels in the Western world and how the ‘green’ environment may help to decrease perception of effort and improve motivation to increase physical activity levels

2. Discuss the impact of green exercise on physiological and psychological markers of health and whether these impacts are enhanced by the green environment

3. Explore the mechanisms that are attributed to green exercise for improvements to health and

4. Discuss the consequences of the disengagement with nature and its impact on health.

### Declining physical activity levels

Worldwide, 31.1% of adults are physically inactive [20]. Some of the decline is attributed to technological advances through the agricultural and industrial revolutions, and more recently, digital revolution. The focus of much structured physical activity in the developed world has also shifted indoors to gymnasia, sports halls, and to within the home; proportionally less physical activity is undertaken outdoors. Due to rapid urbanisation and nearly half of the world’s population living in urban areas [21], less green space and quality green space is available in which undertake physical activity or sport.

### Green spaces, physical activity and health

The decline in physical activity is resulting in huge increases in physical disability and disease [22] and a rising number of cases of mental ill-health [23]. It is essential, therefore, to find ways of engaging all individuals to improve health and prevent further increases in non-communicable diseases. The use of outdoor natural environments for physical activity and health is not new. For 99% of human history, not only we have lived off the land and sought nature for basic survival needs and health, but also for pleasure and physical activity. More recently climbers, hill-walkers, mountain bikers and endurance athletes have all enjoyed the great outdoors and green spaces. It may not only facilitate enjoyment for participants and improve adherence [19], but may also encourage positive physical activity behaviours which are likely to produce greater health gains. As we maybe still genetically designed to be hunter-gatherers in the great outdoors, we are not being stimulated physically or mentally in the same way and this may be detrimental to health. One hypothesis suggests that we are all born with an emotional affiliation for other living organisms, i.e. nature loving [5], which may mean as part of our genetic makeup we are innately predisposed to desire nature contact, and thus maybe green exercise should be used to facilitate physical activity to improve health.

### Green exercise, perception of effort, motivation and behaviour change

Although green exercise is perceived to boost health and this can be used as a powerful extrinsic motivation for exercise, not everyone will be motivated by this. People are motivated to exercise for many different reasons [24]. Some are extrinsically driven by external factors including what others may think of them, whilst others are intrinsically driven, maybe due to the enjoyment or the excitement of the challenge. Others engage for health benefits, whereas some may take part for the social aspect. The promotion of the social and entertainment benefits of physical activity appear to be more successful than those promoting health benefits to persuade individuals to partake in physical activity [25]. Green exercise may help motivation to undertake physical activity by increasing enjoyment and escapism from everyday life, with both a social and entertainment value.

There is even some evidence to suggest that exercise may feel easier when performed in the natural environment. When allowed to self-select walking speed, participants tend actually to walk faster outdoors, compared to indoors. Paradoxically, they report a lower rating of perceived exertion [26].

When asked to reproduce a given level of perceived exertion indoors and outdoors, individuals tend to walk faster at a greater physiological effort (verified by heart rate and blood lactate), suggesting they perceive exercise to be less demanding when performed in the natural environment [27]. A recent paper by members of our research group [28] explored the impact of colour in a video which simulated cycling within a natural environment. Participants cycled for 5 min in three different conditions: an unedited video (predominantly showing green foliage), the same video but with a red filter, and the same video with no colour. Interestingly, despite the video images all being the same apart from the colour, the rate of perceived exertion was decreased in the normal image compared to the other two conditions. Furthermore, total positive mood was increased (as mentioned later in the green exercise and health section). This potentially provides support for the first time that ‘greenness’ is an important component of alterations that are seen. There were no differences in physiological markers, e.g. heart rate and oxygen consumption.

Perception of effort is highly complex, comprising multiple components [29]. Perception of effort during exercise comprises input from the brain and integration of information from the feed-forward centre. The latter, particularly, may be influenced by mood and anxiety. There is also feedback from the various different sensors within the body, including central receptors, e.g. baroreceptors, chemoreceptors, and those within the muscles, e.g. metaboreceptors and mechanoreceptors. These provide physiological and biomechanical information. Input also arises from auditory and visual information. In addition, there will also be the input of cognitive factors like prior experience at a given effort and context of the exercise, e.g. is it training or competition? All of these are integrated pre-consciously and will determine what a participant perceives the effort of exercise to be.

In the case of green exercise, the inputs from the visual system, the feed-forward centre as well as cognitive input may be able to act as a distractive stimulus, reducing the perception of exertion. Indeed this has been suggested for other distractive stimuli e.g. music [30]. It is likely that promoting attention to an external pleasant and green environment reduces awareness of physiologic sensations and negative emotions, thus minimizing the perception of effort. As discussed, mood is enhanced and perception of effort appears to be reduced with greenness [28]. Further evidence is shown, with real and simulated nature, in comparison to other environments (built or indoor) the increasing cognitive components including mood [9,10,12,16,31]. This suggests that green exercise reduces perceived effort and allows individuals to work at higher workloads, which may help to increase the amount of physical activity undertaken and motivation to continue. However, there is a dearth of studies that have investigated whether physical activity levels (duration and frequency) are altered by the exercise environment.

The restorative properties of an environment appear to mediate the frequency of physical activity [19], but most studies focus simply on the relationship between percentage of green space (usually surrounding housing) and physical activity levels. While one European study reported that individuals living in a greener environment were three times more likely to be physically active with a 40% lower chance of being overweight or obese [32], other authors have reported no association between the quantity of immediate green space and self-reported levels of physical activity [33,34]. The limitation of the majority of studies is the paucity of information regarding participants’ actual use of local green space, the perceived quality or even access to green space. Access to green space has been shown to be important for mental health and is associated with longevity and decreased risk of mental illness in Japan [35], Scandinavia [36] and the Netherlands [37]. Access also improves perception of general health [38,39] and quality of life in ageing populations [40]. The quality of the green space may also be associated with health as the biodiversity (i.e. range of species of plants and animals that are present in the environment) enhances the psychological health benefits [41].

It appears that having access to green spaces may facilitate physical activity and thus drive behaviour change by decreasing perception of effort and increasing motivation. An increase in physical activity levels will have a direct impact on health parameters. However, would green exercise offer greater benefits in terms of other markers of health than urban or indoor exercise?

### Green exercise and health

A systematic review of studies comparing indoor versus outdoor activity conducted in natural environment suggests that outdoor activity which is conducted in a natural or green environment causes greater feelings of revitalisation and positive engagement [9]. All types of green exercise activities also improve self-esteem and negative mood subscales, such as tension, anger and depression [42,43]. Interestingly, the first five minutes of green exercise appears to have the biggest impact on mood and self-esteem, suggesting an immediate psychological health benefit [16]. Participating in green exercise activities also affects physiological parameters which differ to the changes observed in matched activity in an urban environment [13]. There are, however, only a handful of studies that have been conducted to investigate physiological health markers [13,14,18,44,45]. Physiological outcomes have included heart rate, blood pressure and autonomic control (using heart rate variability) and endocrine markers including noradrenaline, adrenaline and cortisol (an objective measure of stress).

Post-exercise blood pressure returns to baseline values more quickly after exercising in front of rural scenes compared to urban settings [12]. Japanese studies monitoring the physiological effect of walking within real forest environments (Shinrin-Yoku or forest bathing) reported similar findings. Significantly lower systolic and diastolic blood pressure occurred following both viewing alone and walking in the forest environment when compared to the same activity in an urban environment distinctly lacking in any vegetation or plantation [13,46,47]. A reduction in sympathetic activation assessed by lowered urinary noradrenaline may have elicited these effects on blood pressure [46]. Early work by Ulrich [48] suggested exposure to nature-heightened arousal and attention capacity with observed increases in heart rate. However, viewing scenes of nature following exposure to a stressful video [49] later confirmed that increases in parasympathetic activity occur more synonymous with lowered heart rate. In addition, heart rate variability (HRV), an indicator of autonomic function, increased following both viewing and walking in a forest environment [50]. High HRV suggests healthy autonomic nervous system function and is inversely correlated with cardiovascular disease risk. HRV gives an indication of the adaptability of the nervous system in responding to challenges experienced by an individual such as stress and exercise. Using HRV analysis, a study from Japan [47] showed a tendency for a higher HRV, reflective of parasympathetic activity, whilst participants sat outside within a forest environment. This effect has been repeated indoors in a controlled environment enabling viewing nature alone to be highlighted as a cause for increased parasympathetic activity [14] and decreased heart rate [45].

Increasing the level of psychological stress is detrimental to health, and with stress reportedly increasing [51], methods to help to cope with stress are required. Nature may be one such solution as it does also appear to reduce stress markers. Endocrine markers adrenaline, noradrenaline and the stress hormone cortisol, all fall after being within nature, suggesting that exposure to nature affects the two main stress systems, the sympatho-adrenal medullary and the hypothalamus-pituitary-adrenal axis [13,52]. These studies suggest that exposure to forest environments is relaxing and has stress-reducing properties as observed by reductions in the physiological parameters of blood pressure, heart rate (accompanied by an increase in HRV) and endocrine markers. A further effect associated with the reduction in adrenaline is the improved immune function in the form of increased natural killer cell activity. Natural killer cell activity increased for up to 30 days after a three-day trip to a forest for males but only seven days for females [52]. This suggests that the interaction with nature does not have to be extreme to gain wide-ranging physiological health benefits.

### Connectedness to nature

Parental physical activity behaviours influence not only children’s physical activity patterns but also their attitudes to physical activity and choice of exercise environment. If children engage less with nature, when they become parents their offspring may also be less likely to seek out nature. A cycle of unfamiliarity and disconnectedness is then likely to be passed from generation to generation. The human costs of this separation include attention difficulties and behavioural problems, higher rates of emotional and physical illness and diminished use of the senses [53,54].

Despite evidence suggesting that natural environments facilitate physical activity and provide health benefits [10], relatedness and/or connectedness to nature is declining in particular areas and parts of the world, especially in children and adolescents. This is primarily due to a lack of contact with nature, termed ‘the extinction of experience’ [55] or ‘nature deficit disorder’ [53]. The current generation of youth is largely restricted from accessing nature due to parental fears regarding strangers, traffic and criminal activity [56-58]. Only 10% of today’s generation of youth has regular access to nature, compared to the 40% of adults who did so when they were young [59]. Adolescents living in urbanised areas often perceive the countryside to be intimidating and are reluctant to visit if they have not experienced it as children. The amount of time spent outdoors does appear to be a positive correlate of physical activity in both children and adolescents [60-62]. Although small amounts of time are spent in green space in children, those who do tend to undertake higher intensity activity [63]. However, if generations become disengaged with nature and less importance is placed on the environment as a useful resource for health, the distance to travel to get to the green spaces will increase.

## Discussion

The purpose of this review was to identify and discuss how the great outdoors can benefit the general population. With declining physical activity levels in the developed world, initiatives to curb this downward trend are increasingly important. The great outdoors has been a crucial part of human evolution, and it is likely that this reaches into modern beliefs and attitudes towards nature, both conscious and unconscious [5]. There is evidence to suggest that participating in physical activity in a natural environment, or green exercise, might engage people in physical activity by increasing enjoyment of participation, offering social interaction and increased frequency of activity [19]. Interestingly, participating in green exercise activities alters the perception of effort. For those people engaging in green exercise, the nature element may help achieve a greater intensity of exercise without perception of effort changing. If a person perceives exercise to be easier, it has the potential to be more enjoyable. By reducing the perceived effort experienced during green exercise, a greater intensity may be achieved during the exercise whilst also maintaining adherence to and motivation for the activity. Taken all together, this should help to improve physical activity behaviour. Future studies need to explore the impact of the environment on perception of effort in greater depth, incorporating input from other senses. Furthermore, it needs to examine to what intensity of exercise nature may act as a distracter to perception of effort.

It has been the purpose of previous reviews to assess the role of nature from a health and well-being perspective. There is evidence linking the presence of surrounding green spaces to better physical and mental health. This evidence suggests that better health is impacted by the quality of green space, in particular, by levels of biodiversity. Further research should investigate the importance of biodiversity on health for the careful management of these areas to ensure the maximum benefit for health and for the environment. Considering both the quality of, and access to, green space, evidence for the impact on physical activity is conflicting. Many studies have not explored the use of more distant outdoor spaces for recreational use. The majority of studies use self-reported details of physical activity type, duration and intensity, which is subject to bias. Future studies should therefore use objective methods for assessing both physical activity and exercise environment. Accelerometry and Global Positioning System monitoring including Smartphones should enable this [63].

Engaging in physical activity outdoors provides opportunities linked to better health which is unavailable from indoor activity, such as exposure to sunlight for sufficient vitamin D levels. Additionally, outdoor activity shows greater improvements in mental health compared to indoor activity [9]. Building on the health benefits of outdoor activity, including exposure to nature during outdoor activity, has a synergistic impact on markers of mental well-being and physiological markers [10]. The study to date has identified changes in cardiovascular, endocrine and autonomic function which suggests a psychophysiological impact of nature and green exercise. However, little has been done to identify the mechanisms by which these changes are influenced by experiencing nature. Although seemingly counterintuitive for the purpose of research concerning the great outdoors, the use of controlled indoor environments is important for exploring the alteration of physiological parameters already observed. This will have important implications for using outdoor exercise for rehabilitation or prevention of disease, especially cardiovascular disease.

There are however, disadvantages and barriers to using the great outdoors. How the outdoor space is perceived influences usage. Although individual preferences differ, safety and opportunity for socialization are shown to be the key determinants for use of green spaces [64]. Ease of access, including transportation to the place of interest, suitable links between areas (i.e. footpaths not continuing without crossing/walking down busy roads/private land) all affect participation. Furthermore, socioeconomic status also alters local green space usage for physical activity. Higher socioeconomic status enhances park safety, maintenance, attractiveness and opportunity for socialization and is an important determinant of access to more remote nature (i.e. due to transport required to reach destination). Concerns for personal safety will motivate people to avoid perceived dangerous situations, and going outdoors in some areas does pose a threat. This is influencing parental choices, and there is a growing disconnection with outdoor activity and more specifically nature in the new generation of children. Also, neighbourhood crime safety, aesthetics, and traffic safety all influence participation levels. Safety concerns are also accentuated if the area is remote, where injuries or exposure to the outdoors for a prolonged period of time, especially in extremes of weather may occur. This is predominantly an issue for those individuals who are unprepared, not trained or not supervised correctly.

## Conclusions

To summarise, outdoor natural environments may provide some of the best all-round health benefits by increasing physical activity levels with lower levels of perceived exertion, altering physiological functioning including stress reduction, restoring mental fatigue, and improving mood and self-esteem and perceived health. Thus, exercise within green spaces and the great outdoors may be a useful natural medicine (*vis medicatrix naturae*) [65] to address health challenges facing developed countries. Alongside the social aspect which some individuals crave, it may also increase enjoyment and adherence to bring about positive behaviour changes in a large proportion of the population.

The great outdoors, therefore, should not be just considered a playground for those who seek the thrills of extreme sports, but emphasis should be placed on access for all. One way of doing this is to ensure urban parks are maintained and are developed to produce interesting areas of high biodiversity, as well as more open play areas, where more sports may be played, increasing opportunities for exercise. Not only may both types of area elicit greater health benefits, but also may offer protection for the natural environment and preserve species. The management of countryside, forests and more extreme environments also needs careful consideration including ensuring access for all, but without the pressure of too many people visiting these areas, as this would potentially destroy the natural environment that elicits these health benefits. The challenge for researchers in this field is not only determining whether knowledge of nature’s health benefits can act as a motivator for behaviour change, but also ensuring that the increased use of ‘nature as a therapy’ is accompanied by a conservationist approach to ensure preservation of the environment. It is hoped that by more individuals partaking in green exercise and enjoying the great outdoors, they will retain their evolutionary connection with nature and act to become more protective of it.

## Abbreviation

HRV: Heart rate variability.

## Competing interests

The authors declare that they have no competing interests.

## Authors’ contributions

All authors helped plan, develop and write the manuscript. All authors read and approved the final manuscript.
